# EMSAM: enhanced multi-scale segment anything model for leaf disease segmentation

**DOI:** 10.3389/fpls.2025.1564079

**Published:** 2025-03-14

**Authors:** Junlong Li, Quan Feng, Jianhua Zhang, Sen Yang

**Affiliations:** ^1^ School of Mechanical and Electrical Engineering, Gansu Agricultural University, Lanzhou, China; ^2^ Agricultural Information Institute, Chinese Academy of Agricultural Sciences, Beijing, China; ^3^ National Nanfan Research Institute, Chinese Academy of Agricultural Sciences, Sanya, China

**Keywords:** segment anything model, parameter efficient fine-tuning, adapter tuning, leaf disease segmentation, multi-task learning

## Abstract

Accurate segmentation of leaf diseases is crucial for crop health management and disease prevention. However, existing studies fall short in addressing issues such as blurred disease spot boundaries and complex feature distributions in disease images. Although the vision foundation model, Segment Anything Model (SAM), performs well in general segmentation tasks within natural scenes, it does not exhibit good performance in plant disease segmentation. To achieve fine-grained segmentation of leaf disease images, this study proposes an advanced model: Enhanced Multi-Scale SAM (EMSAM). EMSAM employs the Local Feature Extraction Module (LFEM) and the Global Feature Extraction Module (GFEM) to extract local and global features from images respectively. The LFEM utilizes multiple convolutional layers to capture lesion boundaries and detailed characteristics, while the GFEM fine-tunes ViT blocks using a Multi-Scale Adaptive Adapter (MAA) to obtain multi-scale global information. Both outputs of LFEM and GFEM are then effectively fused in the Feature Fusion Module (FFM), which is optimized with cross-branch and channel attention mechanisms, significantly enhancing the model’s ability to handle blurred boundaries and complex shapes. EMSAM integrates lightweight linear layers as classification heads and employs a joint loss function for both classification and segmentation tasks. Experimental results on the PlantVillage dataset demonstrate that EMSAM outperforms the second-best state-of-the-art semantic segmentation model by 2.45% in Dice Coefficient and 6.91% in IoU score, and surpasses the baseline method by 21.40% and 22.57%, respectively. Particularly, for images with moderate and severe disease levels, EMSAM achieved Dice Coefficients of 0.8354 and 0.8178, respectively, significantly outperforming other semantic segmentation algorithms. Additionally, the model achieved a classification accuracy of 87.86% across the entire dataset, highlighting EMSAM’s effectiveness and superiority in plant disease segmentation and classification tasks.

## 1 Introduction

Accurate segmentation of leaf lesions is essential for the early diagnosis and precise management of crop diseases. The area, shape, and distribution of lesions reflect disease severity and guide subsequent prevention and control measures (Shoaib et al., 2022). Manual segmentation of disease spots by plant pathology experts is time-consuming, labor-intensive, and inefficient. Moreover, this approach is prone to bias and requires significant investment in human and material resources (Singh and Misra, 2017). Computer vision-based methods generally offer better efficiency, consistency, and automation than manual segmentation. They can capture complex feature details, facilitating large-scale processing and analysis. Traditional methods for plant disease segmentation employ edge detection, thresholding, and region growing (Pang et al., 2011; Revathi and Hemalatha, 2012; Wang et al., 2013). These methods are effective for images with simple backgrounds and distinct disease spots. However, they lack robustness when dealing with lesions that have blurred boundaries, complex shapes, and varying sizes.

Deep learning models can automatically learn complex features, showing strong robustness against noise and complex backgrounds, making them efficient for lesion segmentation (Giménez-Gallego et al., 2020). Models based on deep learning are primarily divided into two categories: Convolutional Neural Network (CNN) and Vision Transformer (ViT). CNN-based segmentation models, such as U-Net (Ronneberger et al., 2015) and the DeepLab series (Chen et al., 2018a), achieve precise segmentation of target regions. Liu et al. (2022) used a U-Net model with DenseNet as the backbone to segment three common rice leaf diseases, achieving a mean Dice coefficient (mDice) of 0.86. Yuan et al. (2022) employed an improved DeepLab+ model to segment grape leaf black rot, introducing channel attention mechanisms and pyramid feature fusion networks, resulting in a mean Intersection over Union (mIoU) score of 0.85 on a custom orchard dataset. Pal and Kumar (2023) proposed an AgriDet framework, which integrates the Inception-Visual Geometry Group Network with a Kohonen-based deep learning network to classify the severity of plant diseases. Within this framework, a multi-variate Grab-Cut algorithm is employed to achieve effective image segmentation under complex background occlusion, significantly mitigating the issue of background interference. Divyanth et al. (2023) proposed a two-stage model combining U-Net and DeepLab+. It first extracts leaves from the background, followed by disease spot extraction, achieving an mIoU of 0.74 for corn leaf diseases. Although these works have made progress in leaf disease image detection, CNNs inherently lack global information perception. Pal et al. (2024) proposed a novel framework for plant disease recognition, which initially employs DeepLabV3 to accurately segment the diseased plant regions. Additionally, the framework utilizes Bayesian Task Augmentation-Model Agnostic Meta-Learning with multi-scale spatial attention to optimize the network, enabling the model to achieve exceptional performance even with limited datasets. Experimental results on two datasets demonstrated outstanding performance, with an accuracy rate of 99.1%, a sensitivity of 99.5%, and a specificity of 98.7%. This limits their performance when dealing with leaf disease spots that exhibit distributional differences. Transformer architectures excel at global modeling, which improves segmentation accuracy and helps handle complex disease regions (Dosovitskiy et al., 2021). For instance, Jiang et al. (2023) employed the Trans-Unet model to segment pine nematode disease, employing a novel loss function based on precision and recall, achieving an mDice Coefficient of 0.87. Yang et al. (2024) utilized the Swin-Unet model, optimized with SENet modules to focus on global target features, achieving an mDice Coefficient of 0.85 in corn leaf disease segmentation tasks. However, these methods require extensive annotated data and are less effective in handling blurred boundaries. Additionally, task-specific models require targeted training, limiting their adaptability and generalization to diverse leaf disease segmentation scenarios.

**Figure 1:**
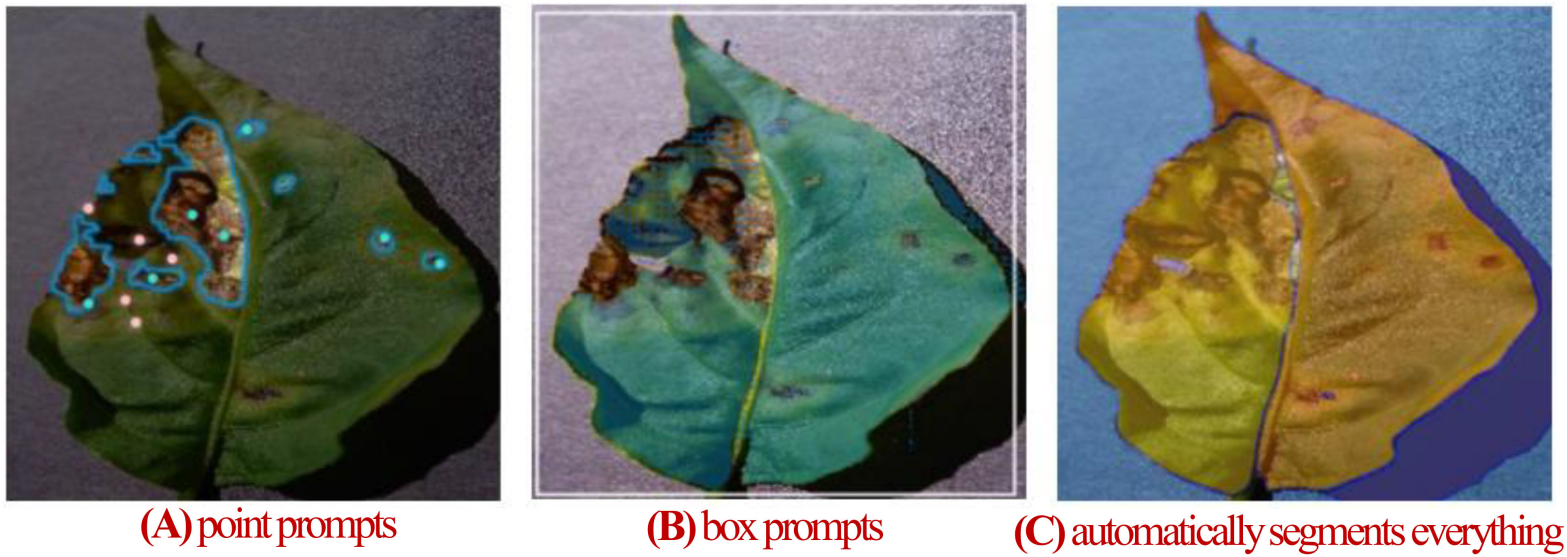
**(A)** Segmentation with SAM’s point prompts. **(B)** Segmentation with SAM’s box prompts. **(C)** SAM automatically segments everything.

Traditional models designed for specific tasks often have limited adaptability and generalization when applied to diverse leaf disease segmentation scenarios. In contrast, the Segment Anything Model (SAM), pre-trained on extensive image datasets, shows exceptional generalization performance. This enables SAM to adapt more effectively and generalize across various leaf disease segmentation tasks. Its efficiency in segmenting targets with both sparse and dense prompts makes it a promising solution for overcoming the limitations of task-specific models, thereby enhancing the versatility and performance in agricultural applications (Kirillov et al., 2023). SAM excels in generalization, performing well in simple natural image segmentation tasks without the need for retraining. However, for specialized tasks such as plant disease segmentation, SAM typically requires fine-tuning to achieve optimal performance (Zhang and Jiao, 2023). Since its release, SAM has garnered significant attention, with applications in medical imaging (Cheng et al., 2023; Wu et al., 2023; Ma et al., 2024), remote sensing (Osco et al., 2023; Wang et al., 2023; Gui et al., 2024), and plant disease segmentation. In the field of plant disease segmentation, Zhang et al. (2023) fine-tuned SAM on a tobacco leaf dataset, achieving an mIoU of 0.84 across different growth stages. Moupojou et al. (2024) employed a two-stage framework where SAM first segments all recognizable objects in an image, and then a self-constructed classification network performs image classification, resulting in a 10% improvement in classification accuracy compared to traditional classification networks. Balasundaram et al. (2025) used SAM to segment diseased tea leaf areas. These segments were processed by a custom CNN for feature extraction, followed by classification, achieving 95.06% accuracy. The above studies use SAM to segment diseases in private datasets, not open datasets, making it difficult to comprehensively evaluate SAM’s segmentation performance on plant diseases. Moreover, Transformer-based SAM faces challenges in capturing fine details, showing limitations in tasks that require fine-grained segmentation, such as blurred boundaries, camouflaged objects, and fragmented features (Zhang et al., 2024). Plant disease images often exhibit these traits, with lesions of low severity showing blurred boundaries and fragmented, fine-grained features. Directly applying SAM to leaf disease segmentation yields unsatisfactory results, as shown in **Figure 1**. **Figure 1A** shows segmentation using point prompts, where foreground points mark disease spots, and background points mark leaves and other areas. However, achieving better results requires precise point settings, significantly reducing efficiency. **Figure 1B** illustrates box prompts, using a bounding box to segment leaves. While box prompts segment the leaf, they fail to isolate disease spots. **Figure 1C** shows automatic segmentation results, distinguishing foreground from background but missing detailed disease regions. resulting masks lack any semantic information. As a result, to enhance SAM’s performance in the leaf disease monitoring field, it often requires fine-tuning with high-quality plant disease segmentation images. This highlights the need for fine-tuning large vision models to adapt them for specific segmentation tasks.

To enable SAM to better learn domain-specific knowledge, parameter-efficient fine-tuning techniques are considered the most effective solutions (Xu et al., 2023). Chen et al. (2023b) proposed SAM-Adapter, integrating domain-specific information or prompts into SAM via efficient adapters. This approach facilitates the adaptation to downstream tasks. Zhang and Liu (2023) proposed SAMed, which fine-tunes SAM’s image encoder using Low-Rank Adaptation (LoRA). This method achieves performance comparable to state-of-the-art semantic segmentation techniques in various medical image segmentation tasks. Li et al. (2023) developed an agriculture-specific SAM adapter, improving the Dice Coefficient by 41.48%. However, these fine-tuning methods still fail to allow SAM to perform effectively with complex features. The main reason for these issues is that plant disease images differ significantly in feature details and distribution from SAM’s training images. The unclear boundary and irregular shape of a lesion in a plant disease image pose significant challenges for SAM. These challenges can be summarized as follows: (1) Leaves with mild disease severity often have indistinct boundaries, resulting in minimal differences between the foreground and background. (2) Lesion areas vary widely, ranging from large clusters to fragmented distributions, or a combination of both. (3) The absence of label information in SAM’s training data prevents the use of individual small lesions for disease type classification. These challenges significantly constrain the model’s ability to effectively segment leaf disease regions with complex features.

In response to these challenges, we propose the Enhanced Multi-Scale SAM (EMSAM), a framework designed to improve SAM’s performance in complex disease segmentation tasks. EMSAM achieves fine-grained segmentation of leaf disease images by focusing on the following key objectives: (1) Adapting the base model specifically for plant disease segmentation. (2) Integrating multi-scale feature modeling to effectively capture lesion characteristics. (3) Combining global feature extraction with local detail capture to improve sensitivity to disease-specific features. (4) Jointly optimizing segmentation and classification in the decoding stage using a unified loss function. The main contributions of this study include:

1. Development of the EMSAM framework, which integrates more efficient adapter tuning techniques to enhance its performance in plant disease segmentation. The framework improves SAM’s ability to handle disease images with blurred boundaries and complex shapes.
2. Design of the Multi-Scale Adaptive Adapter (MAA): The MAA module captures multi-scale pyramid features to extract disease features at various scales, improving the model’s segmentation precision and robustness. This is achieved with minimal trainable parameters, enabling efficient parameter tuning.
3. Introduction of an efficient feature fusion mechanism: Combining a Local Feature Extraction Module (LFEM) with the Feature Fusion Module (FFM), EMSAM incorporates cross-branch and channel attention mechanisms to optimize the integration of CNN and ViT features, achieving a balance between global and local feature representation.
4. Incorporation of a lightweight classification head and a joint loss function: This enables EMSAM to accurately segment lesion regions while predicting disease categories, significantly enhancing the practical utility of the model.
5. We conduct the first comprehensive evaluation of SAM and EMSAM on the PlantVillage dataset, which is the well-known open plant disease image dataset, establishing a comparable baseline for future research in plant disease segmentation.

The remainder of this paper is organized as follows: Section 2 provides a detailed description of the dataset construction and EMSAM architecture. Section 3 describes the experimental setup and analyzes the results. Section 4 discusses the implications and significance of the study and summarizes the overall research.

## 2 Materials and methods

### 2.1 Dataset and construction method

The dataset employed in this study is the widely recognized PlantVillage dataset, which is extensively utilized in the field of plant disease segmentation (Hughes and Salathe, 2016). Comprising a total of 54,306 plant leaf images, the dataset is organized into 12 categories of healthy leaves and 26 categories of diseased leaves. Notably, all disease types have been diagnosed by plant pathology experts, ensuring the accuracy and reliability of the data. The images are in RGB format, with a uniform resolution of 256x256 pixels, facilitating consistent preprocessing and analysis.

We employ the “EISeg” tool (Hao et al., 2022) from Baidu’s PaddlePaddle framework for pixel-level annotations of disease lesion areas, saving the annotated data in PNG format. For our study, we annotated 200 images per category across 26 diseased leaf categories, following these criteria:

1. Diversity of Lesion Characteristics: Images were selected to capture variations in lesion shapes, sizes, color patterns, and spatial distributions, ensuring representation of early to late disease stages.
2. Class Balance: Each category was strictly limited to 200 images to prevent model bias toward overrepresented classes.

The resulting dataset, named the PlantVillage Segmentation Dataset (PSD), contains 5,200 images (26 classes × 200 images) split into a training set (4,160 images) and a test set (1,040 images) with an 8:2 ratio. **Table 1** details the category distribution and annotation statistics.

**Table 1:** Category information of the PSD.

Class ID	Class name	Class ID	Class name
1	Apple scab	14	Potato early blight
2	Apple black rot	15	Potato late blight
3	Apple cedar rust	16	Squash powdery mildew
4	Cherry powdery mildew	17	Strawberry leaf scorch
5	Corn cercospora leaf spot	18	Tomato bacterial spot
6	Corn rust	19	Tomato early blight
7	Corn northern leaf blight	20	Tomato late blight
8	Grape black rot	21	Tomato leaf mold
9	Grape black measles	22	Tomato septoria leaf spot
10	Grape leaf blight	23	Tomato spider mites
11	Orange citrus greening	24	Tomato target spot
12	Peach bacterial spot	25	Tomato mosaic virus
13	Pepper bacterial spot	26	Tomato yellow leaf curl

**Figure 2:**
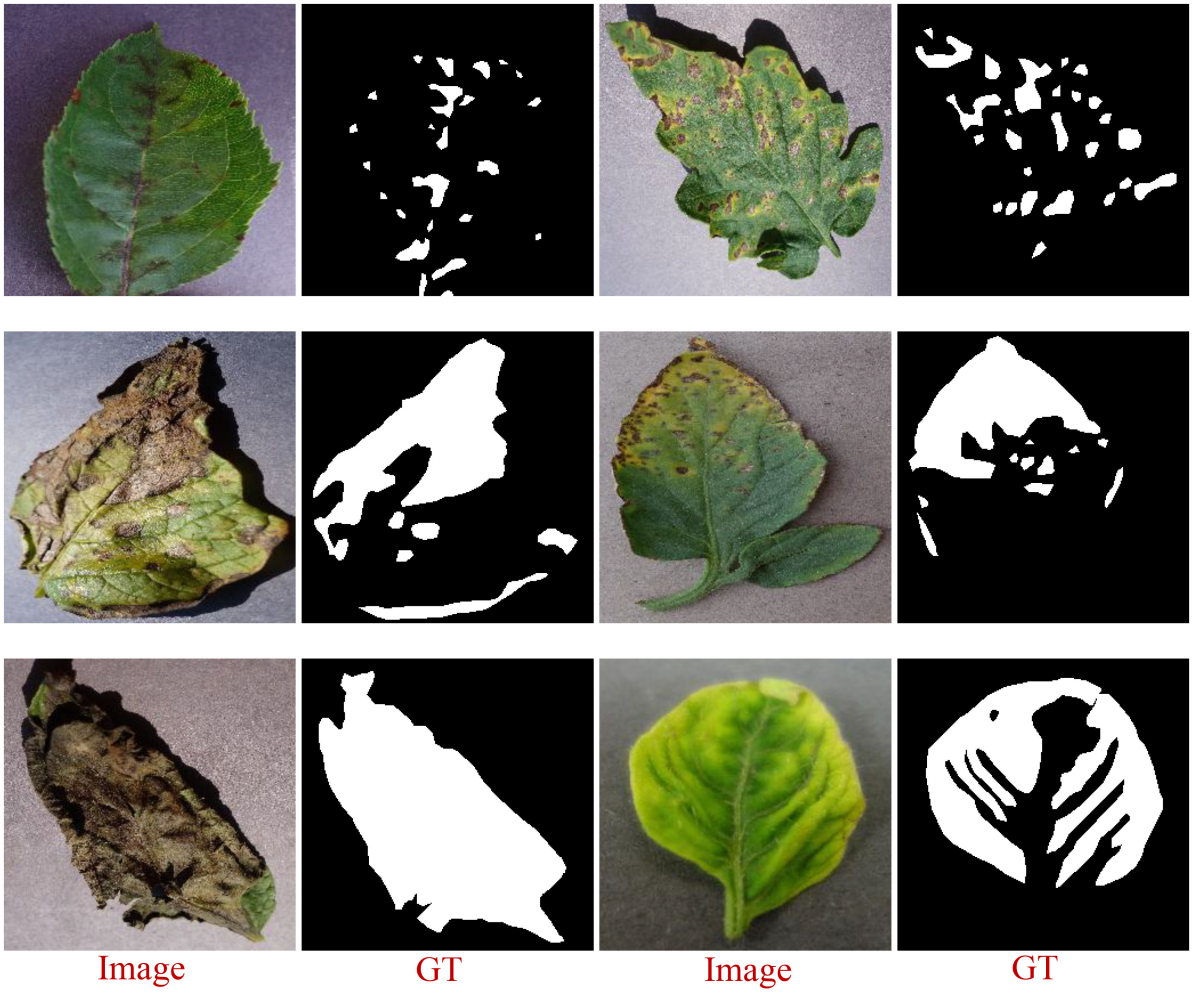
A selection of images showing areas of lesions labeled with EISeg.

The PSD includes images displaying various lesion distributions: some with small, fragmented lesions; others with large, concentrated lesions; and some exhibiting a combination of both characteristics. These complex shapes reflect the irregularity of lesion area distributions on leaves, posing higher demands on models tasked with leaf disease segmentation. **Figure 2** illustrates selected annotated images from the PSD, showcasing original images alongside their corresponding grayscale ground truth masks. The first-row features images with fragmented disease spot distributions, the second row shows images with mixed fragmented and concentrated distributions, and the third row displays images with concentrated disease spots.

To demonstrate the model’s capability in segmenting leaves with varying levels of disease severity, we adopt the leaf disease severity classification method proposed by Ji and Wu (2022), utilizing the Percentage of Infections ( POI ) as a baseline metric. The infection percentage is calculated using Equation 1:


(1) $$\begin{array}{l} POI=(D_{a}/T_{a})\times 100\\ \;=(P_{i}/P_{t})\times 100 \end{array}$$

Where $D_{a}$ and $T_{a}$ denote the mutilated leaf area and the total leaf area, respectively, $P_{i}$ and $P_{t}$ denote the pixel size of the diseased spot area and the pixel size of the total leaf area, respectively. Based on this metric, disease severity is categorized into three levels: light (0< POI ≤ 0.2), moderate (0.2< POI ≤ 0.5), and severe (0.5< POI ≤ 1). Utilizing these three severity levels, we further subdivide the test set to facilitate more detailed experimental validation.

### 2.2 Overall architecture of EMSAM

**Figure 3:**
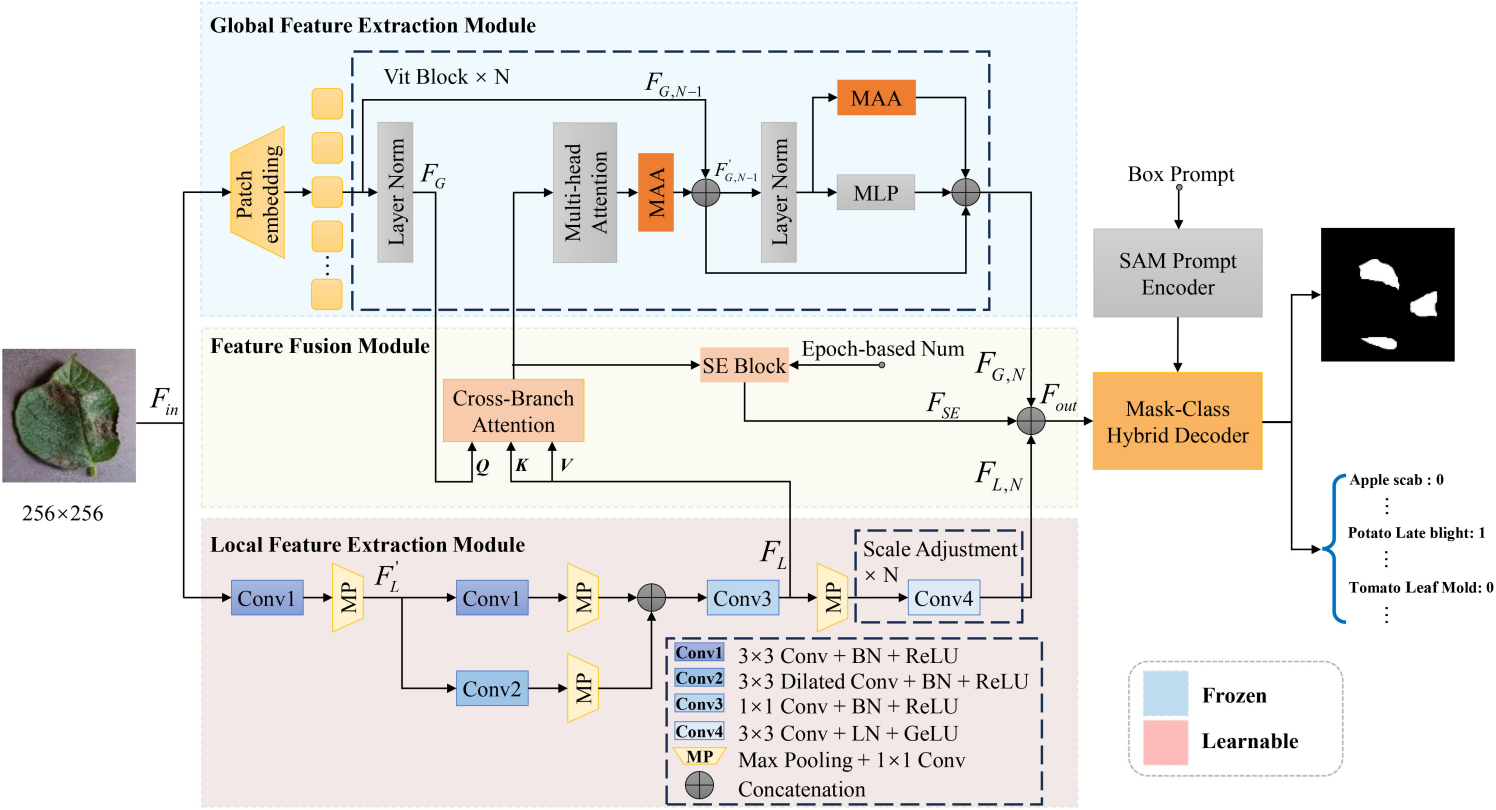
Overall architecture of the proposed EMSAM. It enhances the multi-scale fine-grained image processing capability of SAM through four novel modules: MAA, LFEM, FFM, and mask-category hybrid decoder.

For the task of plant leaf disease segmentation and classification, we propose the Enhanced Multi-scale SAM (EMSAM), as illustrated in **Figure 3**. The EMSAM architecture comprises three primary components: an image encoder, SAM’s prompt encoder, and a hybrid decoder. The image encoder is responsible for fusing global and local features, SAM’s prompt encoder is responsible for generating the prompt embedding, and the hybrid decoder concurrently handles classification and segmentation tasks. The image encoder consists of two branches: a ViT branch forming the Global Feature Extraction Module (GFEM) and a CNN branch comprising the Local Feature Extraction Module (LFEM). The GFEM employs stacked ViT blocks to extract global features, with most parameters frozen during training. Only the parameters related to the MAA integrated into the ViT blocks are fine-tuned. The LFEM leverages convolutional modules and multi-scale feature extraction techniques to capture local features, thereby enhancing sensitivity to edges and textures. The global and local features are subsequently fed into the FFM. The FFM employs cross-branch attention mechanisms and progressively integrated Squeeze-and-Excitation (SE) blocks (Hu et al., 2018) to facilitate effective interaction and weighted fusion, resulting in comprehensive feature representations. In the decoding phase, the Mask-Class Hybrid Decoder integrates the fused features from the image encoder with the prompt embeddings from SAM’s Prompt Encoder. A lightweight linear layer acts as the classification head, responsible for predicting mask confidence, Intersection over Union (IoU) tokens, and label identifiers.

#### 2.2.1 Multi-scale adaptive adapter in ViT block

**Figure 4:**
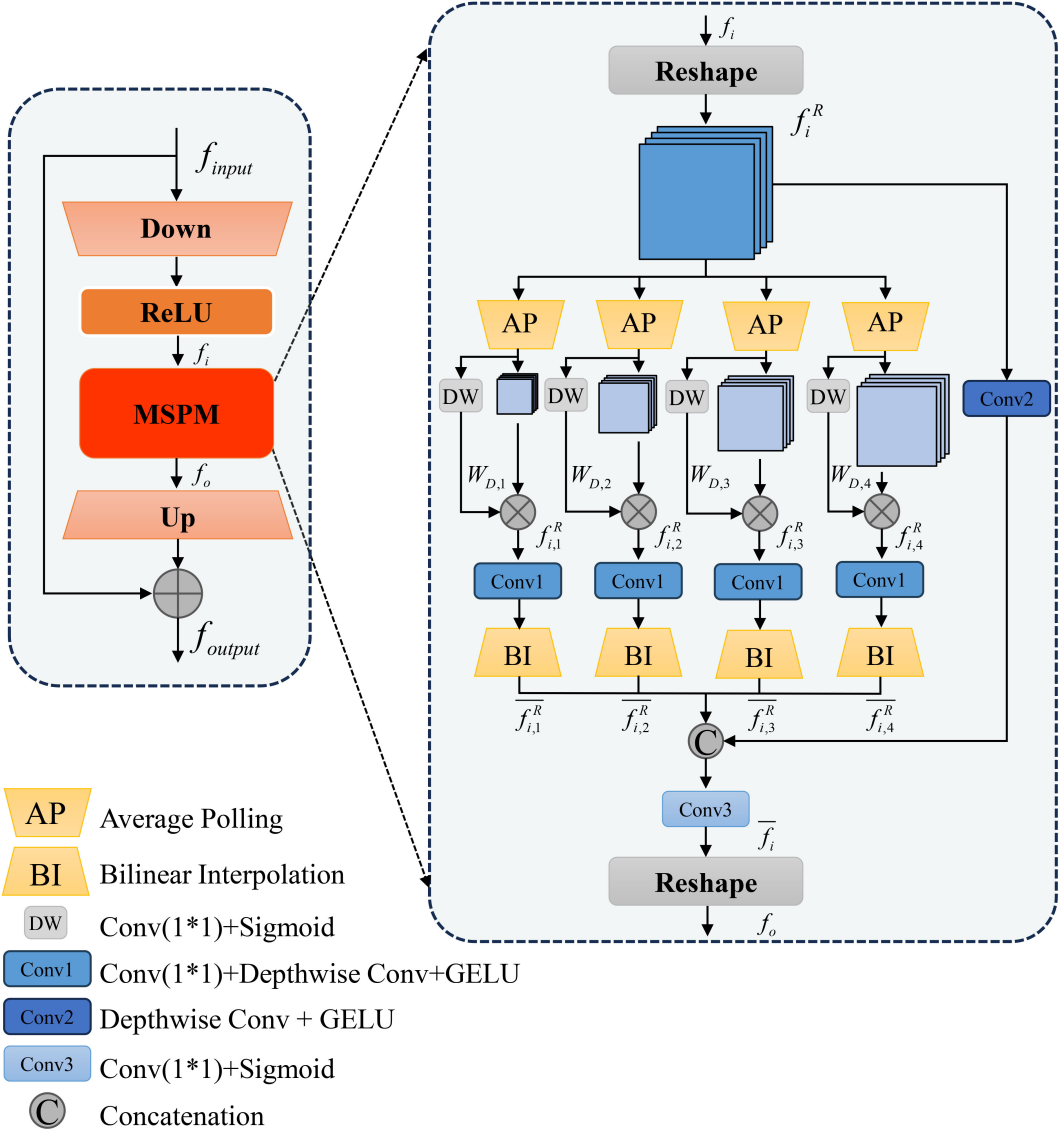
Detailed structure of the proposed MAA module.

Although SAM excels in natural scene segmentation, it necessitates fine-tuning for specific downstream tasks. The image encoder of SAM, which employs stacked ViT blocks, results in a large number of trainable parameters, thereby limiting performance and increasing the risk of overfitting, particularly when training data are scarce. To address these challenges, adapter tuning offers an efficient and cost-effective approach to adapt pre-trained models (Sung et al., 2022). Consequently, we design the MAA to efficiently adapt SAM for the leaf disease image domain. The detailed architecture of MAA is illustrated in **Figure 4**.

Within the GFEM, each ViT Block comprises a multi-head attention mechanism and an MLP layer, with layer normalization applied before each sublayer. In our architecture, we insert two MAAs into each ViT Block as trainable parameters, while freezing the remaining components to preserve the pre-trained weights. These MAAs incorporate depthwise separable convolutions, enabling a lightweight design that significantly reduces the training cost while enhancing the model’s adaptability to the specific task of leaf disease segmentation.

Traditional Adapter structures include a down-sampling linear layer (Down), a ReLU activation function, and an up-sampling linear layer (Up). In our design, to obtain multi-scale features and optimize adapter-tuning, we add a Multi-Scale Pyramid Feature Module (MSPM) after the ReLU activation function. Thus, the adapter tuning process can be represented by Equation 2:


(2) fiR=τ(UP(MSPM(ReLU(Down(finput)))))

Where $f_{input}$ denotes the input features, which are sequentially processed through a linear down-sampling layer and a ReLU activation function. The processed features are then transformed into $f_{i}^{\text{R}}\in$ ${\text{ℝ}}^{\frac{D}{r}\times W\times H}$ within the Multiscale Pyramid Feature Module (MSPM) for subsequent multi-scale spatial information processing, while r represents a reduction factor introduced to decrease the dimensionality of the input features. τ denotes the reshaping operation applied to the input features, where the global features are decomposed into multiple sub-features to enable parallel processing of information at different scales or across distinct regions.

In the MSPM, to enhance the utilization efficiency of multi-scale features, we employ four global average pooling layers (AP) to extract multi-scale features, denoted as $f_{i,j}^{\text{R}}\in$ ${\text{ℝ}}^{\frac{D}{4\times r}\times W\times H}$ . Subsequently, simple 1×1 convolution layers are designed to generate dynamic weights $W_{D,j}$ . These dynamic weights allow each input feature to dynamically adjust the importance of each scale based on its content, effectively avoiding the fixed contribution of features at each scale. This process is formally represented by Equations 3, 4, and 5:


(3) $$W_{D,j}=DW(AP(f_{i}^{R})),1\leq j\leq 4$$

(4) $$f_{i,j}^{R}=AP(f_{i}^{R})\cdot W_{D,j},0<W_{D,j}<1$$

(5) $$\bar{f_{i,j}^{R}}=BI(C_{1}(f_{i,j}^{R}))$$

Where the dynamic weights $W_{D,j}\in \left(0,1\right)$ are constrained within a reasonable range using the Sigmoid activation function. $C_{1}$ defines a convolutional layer with a kernel of 1×1 and a kernel of 3×3 depthwise separable convolution with GELU activation function. BI is a bilinear interpolation-based upsampling method that restores downsampled features to their original resolution.

Next, we concatenate the multi-scale features $\bar{f_{i,j}^{\text{R}}}\in$ ${\text{ℝ}}^{\frac{D}{r}\times W\times H}$ , representing the processed multi-scale spatial information in Equation 6:


(6) $$\bar{f_{i}}=C_{3}(\left[\bar{f_{i,1}^{R}},\bar{f_{i,2}^{R}},\bar{f_{i,3}^{R}},\bar{f_{i,4}^{R}},C_{2}(f_{i}^{R})\right])$$

Where $C_{2}$ defines a 3×3 depthwise separable convolutional layer with a GELU activation function, optimizing the overall feature information extraction capability. [·] represents the process of channel-wise concatenation of features from different scales. $C_{3}$ defines a 1×1 convolutional layer, which is applied to impose weight control on the channel dimension of the output feature, acting on the concatenated feature map and facilitating the fusion of global channel weights.

Finally, the entire MSPM output is mathematically represented by Equation 7:


(7) $$f_{o}=\tau \left[\bar{f_{i}}\right]$$

Where τ[·] similarly represents the operation of reshaping the features to match the input feature dimensions. This design enables EMSAM to fine-tune the ViT blocks efficiently, minimizing the training cost.

#### 2.2.2 Enhanced detail feature image encoder

**Figure 5:**
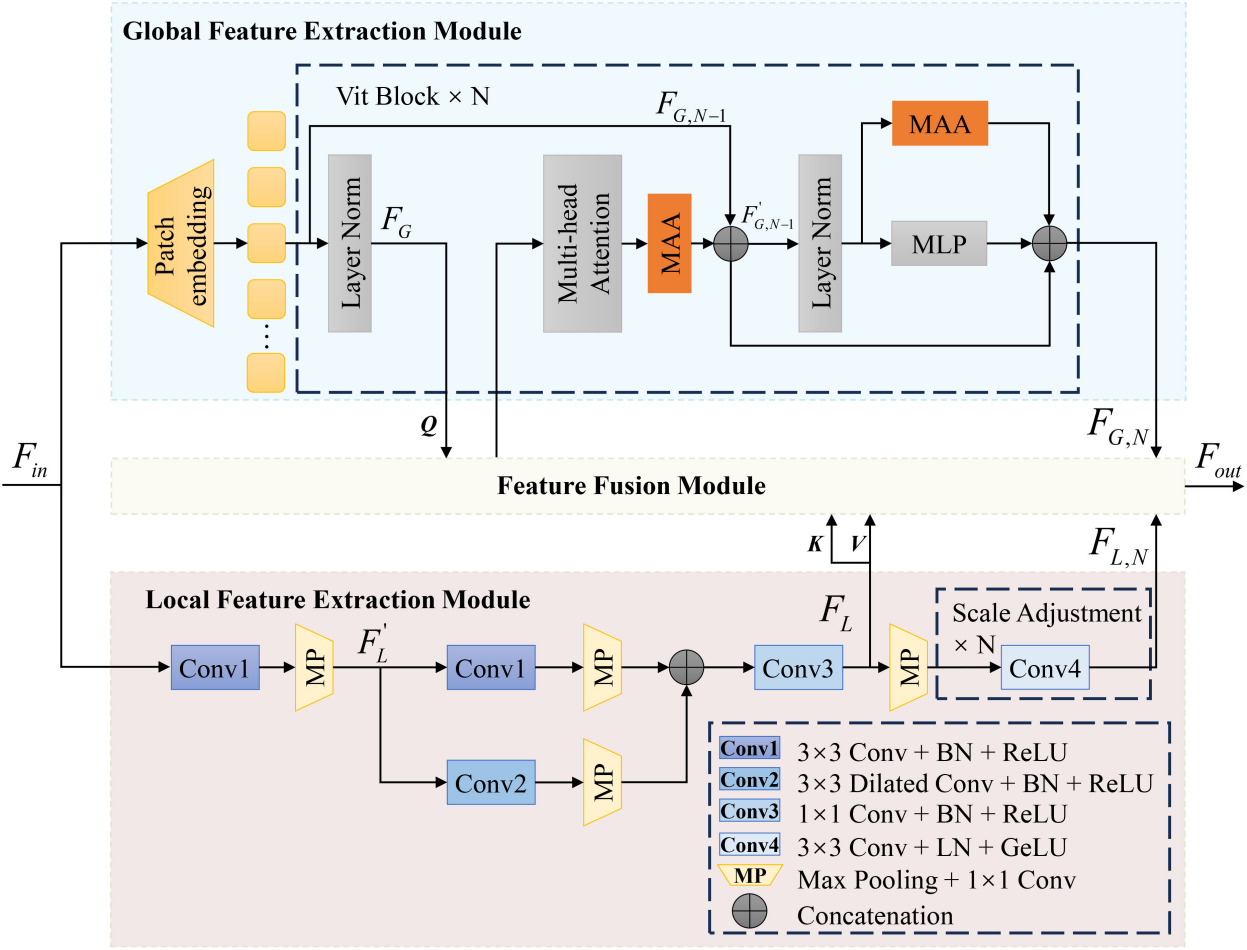
Image encoder with enhanced detail features.

Traditional segmentation models relying exclusively on CNN or ViT often face challenges in effectively capturing both global and local information, particularly when dealing with the complex and irregular feature distributions characteristic of leaf disease images (Ngo et al., 2024). To address these limitations, we introduce the Enhanced Detail Feature Image Encoder, as depicted in **Figure 5**. This encoder consists of two key components: the GFEM, designed to capture global contextual information, and the LFEM, focused on extracting boundary and detailed features. By integrating these modules, the encoder significantly enhances the model’s feature extraction capabilities, thereby improving segmentation performance.

In the GFEM, we inherit the ViT framework from SAM and incorporate the MAA in each ViT Block. The MAA module, through depthwise separable convolutions and the multi-scale pyramid feature module (MSPM), optimizes the ViT’s ability to capture global information specific to leaf disease images. This process can be expressed by Equation 8:


(8) foutput=MAA(finput)        =Up(MSPM(ReLU(Down(finput)))

Additionally, the GFEM leverages multi-head self-attention mechanisms to effectively aggregate critical information globally, enabling the model to capture the overall characteristics of diseased leaves. In the N-th ViT Block, the entire process is given in Equations 9, 10 and 11:


(9) $$F_{G}=LN(F_{G,N-1})$$

(10) $$F_{G,N}^{'}=MAA(Attention(FFM(F_{G})))+F_{G,N-1}$$

(11) $$F_{G,N}=MLP_{SAM}(LN(F_{G,N}^{'}))+MAA(LN(F_{G,N}^{'}))+F_{G,N}^{'}$$

Where $F_{G}$ denotes the input to the FFM , $F_{G,N}$ and $F_{G,N-1}$ represent the output features of the *N*-th and (*N-1*)-th ViT Blocks, respectively, $F_{G,N}^{'}$ represents the intermediate parameters within the ViT Block, and FFM denotes the feature fusion module.

In the LFEM, a multi-layer convolutional structure comprising four convolutional groups and a max-pooling convolution group is employed to precisely capture detailed features. The input features first undergo a 3×3 convolution to extract core features while maintaining computational stability. A parallel branch incorporates a dilated convolution to expand the receptive field and capture larger-scale local features. Post addition, a 1×1 convolution reduces computational load through channel compression and performs nonlinear feature mapping. Each convolutional layer is followed by a Batch Normalization layer to normalize outputs, thereby accelerating the training process and enhancing model performance and stability. ReLU activation functions are applied to all convolutional layers. To match the spatial resolution of the GFEM’s output feature maps, the final stage employs N 3×3 convolutions with Layer Normalization and GELU activation. This process is illustrated in Equations 12, 13, and 14:


(12) $$F_{L}^{'}=MP(Conv_{1}(F_{in}))$$

(13) $$F_{L}=Conv_{3}(MP(\sum_{n=1}^{2}Conv_{n}(F_{L}^{'})))$$

(14) $$F_{L,N}=Conv_{4}(MP(F_{L}))$$

Where $F_{L}$ represents the portion of the LFEM features input to the FFM. $F_{L}^{'}$ denotes the intermediate features during the processing stage. MP refers to the max-pooling operation, which is used to downsample feature maps while retaining prominent local features. Notably, $F_{L,N}$ and $F_{G,N}$ maintain identical spatial resolutions. Thus, the combined output from the image encoder can be expressed by Equation 15:


(15) $$F_{out}=F_{G,N}+F_{L,N}$$

#### 2.2.3 Feature fusion module

**Figure 6:**
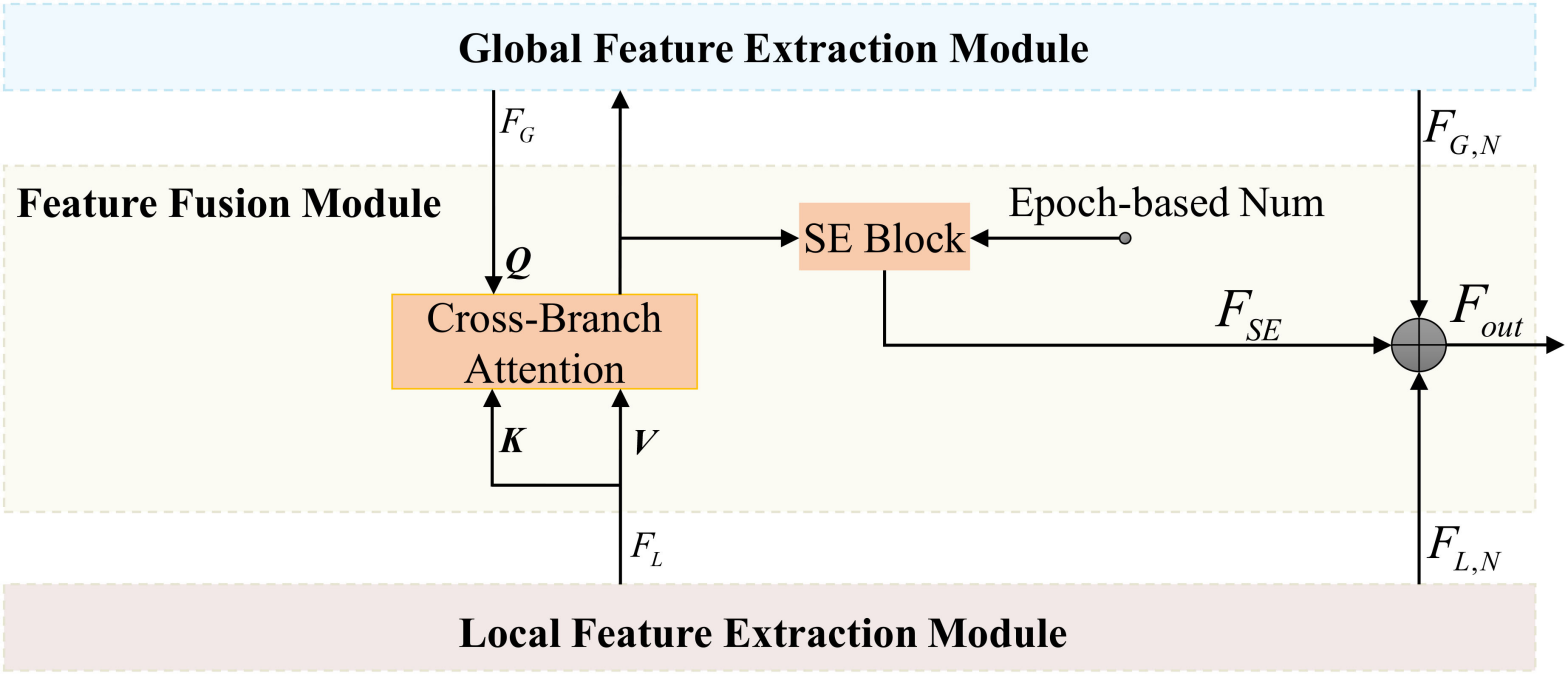
Architecture of the FFM.

To effectively integrate global and local features from the image encoder, we designed the Feature Fusion Module (FFM), as depicted in **Figure 6**. This module balances global and local features, enabling the model to learn the diverse distributions of disease spots in leaf images. To enhance feature flexibility, we incorporated a SE Block into the FFM. The SE Block adjusts channel-wise feature weights, emphasizing key features. The weight adjustment progressively increases over training epochs, guided by an epoch-based scaling factor, ensuring the model adapts to the influence of the SE Block.

In the FFM, the Cross-Branch Attention (CBA) replaces the Multi-head Attention in the original transformer block. These two are essentially the same in nature, except that the keys (*K*) and values (*V*) input to the CBA are derived from the local feature extraction module. To emphasize the interaction between different branches, we refer to this module as Cross-Branch Attention. We first apply CBA to interact between global features $F_{G}$ and local features $F_{L}$ . Using query (Q), key, and value, CBA uncovers effective complementary information across different branches, given in Equations 16 and 17:


(16) $$Q=W_{q}F_{G},\;K=W_{k}F_{L},\;V=W_{v}F_{L}$$

(17) $$CBA(Q,K,V)=Softmax\left(\frac{QK^{T}}{\sqrt{d}}\right)V$$

Where $W_{q}$ , $W_{k}$ , and $W_{v}$ are the learnable weight matrices, while d represents the feature dimension used to scale the inner product.

For the initially interacted features, we employ the SE Block to implement dynamic weight adjustment. The SE Block dynamically adjusts the importance of global and local features based on training epochs, enhancing the adaptability and robustness of the model’s information processing. The operations of the SE Block can be expressed by Equations 18 and 19:


(18) $$S=\sigma (W_{2}\delta (W_{1}z)\cdot \alpha_{epoch})$$

(19) $$F_{SE}=S\cdot CBA(Q,K,V)$$

Where z represents the global average pooling result of the feature vector. σ and δ denote the Sigmoid and ReLU activation functions, respectively. $W_{1}$ and $W_{2}$ correspond to the weights of fully connected layers. $\alpha_{epoch}\;$ is a dynamic coefficient associated with the training epochs, controlled by an incremental function in the form of exponential growth, expressed as follows:


(20) $$\alpha_{epoch}=1-e^{-\beta \cdot current\;epoch}$$

In the above equation, current epoch represents the current training epoch, and β is a hyperparameter controlling the growth rate.

#### 2.2.4 Mask-class hybrid decoder

**Figure 7:**
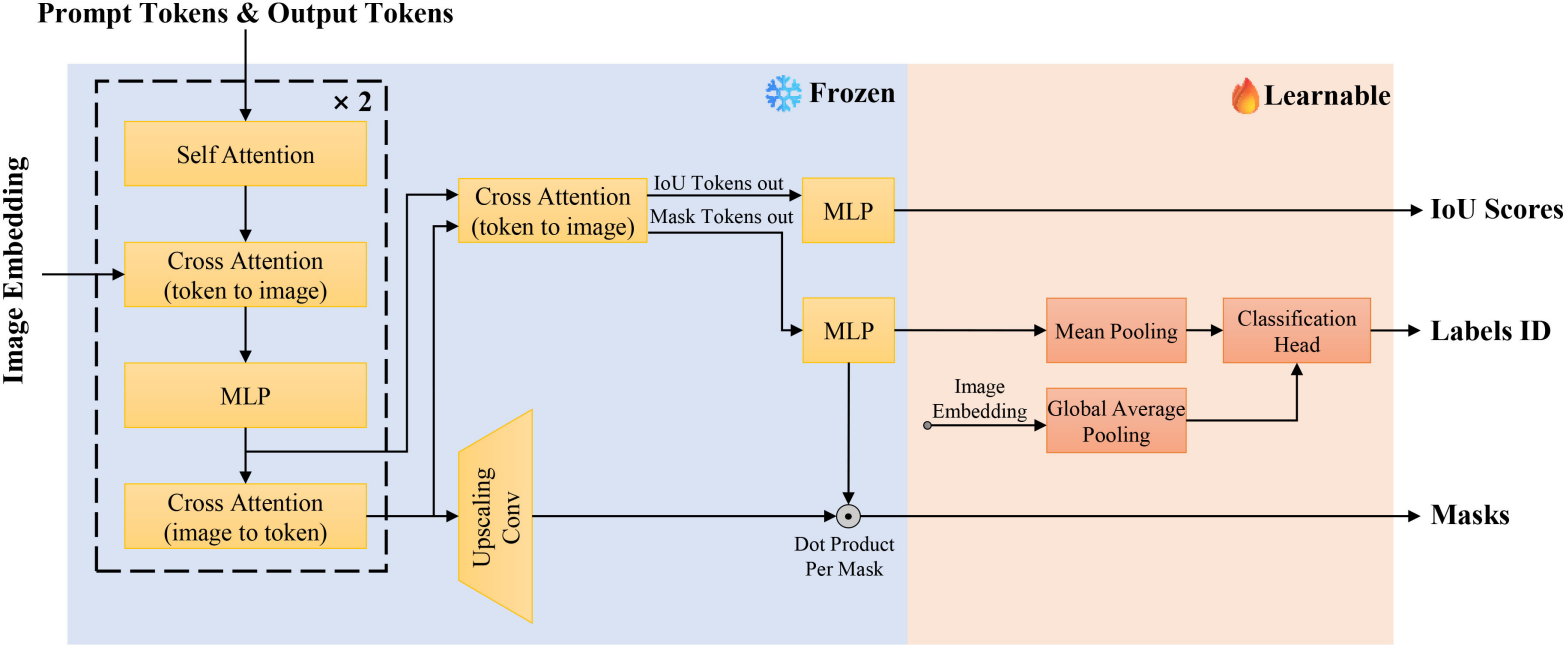
Architecture of the Mask-class hybrid decoder.

Although the SAM mask decoder effectively generates segmentation masks through multi-layer cross-attention mechanisms, it lacks the ability to provide class-specific predictions. To enhance disease segmentation efficiency, which requires both precise lesion delineation and accurate disease type classification, we introduce the Mask-Class Hybrid Decoder. This architecture integrates classification functionalities into the SAM mask decoder, as shown in **Figure 7**.

The SAM mask decoder utilizes self-attention and bidirectional cross-attention to extract interactive features between image and prompt embeddings. These features are processed by an upsampling convolution module to produce binary segmentation masks. However, this approach does not offer class-specific predictions. To address this, we propose adding a lightweight classification head to the existing mask decoder architecture. This head consists of two fully connected layers preceded by a global average pooling layer for dimensionality reduction. By projecting the image embeddings through these layers, we obtain class probability distributions corresponding to the generated segmentation masks. The lightweight design ensures minimal additional training overhead while enabling concurrent classification tasks effectively.

### 2.3 Loss function and model evaluation metrics

#### 2.3.1 Loss function

To simultaneously optimize the performance of segmentation and classification tasks, we define a joint loss function ${ℒ}_{joint}$ , which comprises three components: segmentation loss ${ℒ}_{mask}$ , IoU loss ${ℒ}_{IoU}$ , and classification loss ${ℒ}_{cls}$ . These components are linearly combined as follows:


(21) $${ℒ}_{joint}=\lambda_{mask}\cdot {ℒ}_{mask}+\lambda_{IoU}\cdot {ℒ}_{IoU}+\lambda_{cls}\cdot {ℒ}_{cls}$$

Here, $\lambda_{mask}$ , $\lambda_{IoU}$ , and $\lambda_{cls}$ are the weights assigned to each loss component.

The segmentation loss ${ℒ}_{mask}$ serves as the primary objective function, focusing on the precision of boundary delineation and the consistency of segmented regions. We adopt a combination of Dice Loss and Binary Cross-Entropy (BCE) Loss for the segmentation loss, optimizing both the overlap rate of target regions and pixel-level classification accuracy, as illustrated in Equation 22:


(22) $${ℒ}_{mask}=\alpha \cdot {ℒ}_{Dice}+\beta \cdot {ℒ}_{BCE}$$

Dice Loss emphasizes the overlap between the target region and the prediction, addressing the imbalance between foreground and background areas, which is suitable for segmentation tasks like lesion segmentation where the foreground area is relatively small. BCE Loss measures the correctness of each pixel classification, suitable for scenarios where the foreground and background are relatively balanced. Dice Loss and BCE Loss are defined below, respectively, as shown in Equations 23 and 24:


(23) $${ℒ}_{Dice}=1-\frac{2\sum_{i=1}^{N}y_{i}\hat{y_{i}}}{\sum_{i=1}^{N}{\left(y_{i}\right)}^{2}+\sum_{i=1}^{N}{\left(\hat{y_{i}}\right)}^{2}}$$

(24) $${ℒ}_{BCE}=-\frac{1}{N}\sum_{i=1}^{N}\left[y_{i}\log\left(\hat{y_{i}}\right)+(1-y_{i})\log\left(1-\hat{y_{i}}\right)\right]$$

Where $\hat{y_{i}}$ and $y_{i}$ represent the predicted value and the ground truth value of the *i*-th pixel, respectively.

IoU loss ${ℒ}_{IoU}$ aims to further enhance the model’s prediction of the overlap ratio between the predicted and ground truth regions, effectively focusing on boundary information. This process is illustrated in Equation 25:


(25) $${ℒ}_{IoU}=1-\frac{\left|P\cap G\right|}{\left|P\cup G\right|}$$

Where P represents the number of pixels in the predicted region, G denotes the number of pixels in the ground truth region, |P∩G| and |P∪G| correspond to the number of pixels in the intersection and union of the predicted and ground truth regions, respectively.

The classification loss ${ℒ}_{cls}$ extends the capability of the segmentation task by optimizing the prediction of class labels through cross-entropy loss, as shown in Equation 26:


(26) $${ℒ}_{cls}=-\sum_{i=1}^{C}x_{i}\log(\hat{x_{i}})$$

Where C is the number of classes, $x_{i}$ and $\hat{x_{i}}$ are the true label and the predicted label for class c, respectively.

#### 2.3.2 Model evaluation metrics

To evaluate the performance of EMSAM in leaf disease image segmentation and classification tasks, we employ three categories of evaluation metrics: Dice Coefficient (Dice), Intersection over Union (IoU), and Accuracy (Acc).

The Dice is a metric used to measure the overlap between the predicted segmentation and the ground truth, reflecting the model’s ability to accurately predict the foreground regions. The expression is given by Equation 27:


(27) $$Dice=\frac{2\cdot \left|P\cap G\right|}{\left|P\right|+\left|G\right|}$$

The IoU is a metric that measures the ratio of the intersection to the union between the predicted results and the ground truth segmentation. Compared to the Dice Coefficient, IoU places greater emphasis on strict matching of boundary regions, making it suitable for evaluating the model’s precision in delineating the boundaries of diseased areas. The equation is given by Equation 28:


(28) $$IoU=\frac{\left|P\cap G\right|}{\left|P\cup G\right|}$$

Acc refers to the proportion of correctly classified labels for a given instance averaged over the total number of labels (including both predicted and actual values). The calculation equation is given by Equation 29:


(29) $$Acc=\frac{TP+TN}{TP+TN+FP+FN}$$

Where TP (True Positives) and TN (True Negatives) represent correctly predicted positive and negative instances, respectively, while FP (False Positives) and FN (False Negatives) represent incorrectly predicted instances.

By employing these metrics, we can comprehensively assess both the segmentation and classification performance of the EMSAM model.

## 3 Results and analysis

### 3.1 Experimental setup

The hardware configuration for our experiments is as follows: CPU - Intel^®^ Core™ i7-13700KF @ 5.4GHz, GPU - NVIDIA GeForce RTX 3090 (24GB), and memory - 32GB Samsung DDR5 5600MHz (16GB×2). The experimental environment is set up on an Ubuntu 20.04.6 LTS 64-bit operating system, using Python 3.11 as the programming language, PyTorch 2.0.1 as the deep learning framework, CUDA Toolkit version 12.2, and cuDNN version 8.9.0.

During training, the primary parameter settings are as follows: we select the ViT-b image encoder as the pre-trained model, with an input image size of 256×256 pixels. The patch size for the patch embedding block is set to 16, and the windowed attention size is 14×14. The batch size is set to 4, and the total number of training epochs, including those for comparative experiments, is 200. The training process utilizes the AdamW optimizer with an initial learning rate of 0.0005, adopting an exponential decay learning rate scheduling strategy. The learning rate at the t -th iteration is defined as: $lr(t)=lr_{0}\cdot exp(-kt)$ . Where lr(t) refers to the learning rate at the t -th iteration, $lr_{0}$ is the initial learning rate at the start of training, k is the decay rate constant, and t represents the current training iteration number. This setup facilitates rapid convergence in the early stages of training, helping the model quickly locate a favorable region in the parameter space and avoid over-reliance on local minima. As training progresses, the learning rate gradually decreases, reducing the step size of model updates, which aids in fine-tuning model parameters and effectively prevents oscillations and overfitting in the later stages of training.

### 3.2 Comparison with state-of-the-art methods

We compare EMSAM with six other models to evaluate its effectiveness in segmenting diseased leaf regions. The models include SAMUS (Lin et al., 2024) and MedSAM (Ma et al., 2024) (SAM extensions), Swin-Unet (Cao et al., 2023) and Trans-Unet (Chen et al., 2021) (ViT-based), DeepLabv3+ (Chen et al., 2018b) (ResNet-based), and HRNet-48 (Wang et al., 2021) (HRNet-based). The comparison focuses on three key metrics: Dice coefficient, IoU score, and the backbone networks utilized by each model. These metrics collectively assess the feature extraction and segmentation effect of each model. The quantitative experimental results are presented in **Table 2**.

**Table 2:** Experimental results comparing EMSAM and SOTA methods.

Method	Backbone	Dice	IoU
SAMUS	CNN + SAM	0.7076	0.6336
MedSAM	SAM	0.6448	0.5134
DeepLabv3+	Resnet-50	0.7730	0.6504
Swin-Unet	Swin Transformer	0.5395	0.4483
Trans-Unet	Vit-b	0.5882	0.4984
HRNet-48	HRNet-48	0.6911	0.5643
EMSAM	CNN + SAM	**0.7925**	**0.6987**

The bold values indicate the optimal data metrics achieved by the model under the current experimental setup.

From **Table 2**, it is evident that EMSAM outperforms all other models on the PSD. Leveraging deep CNN-ViT feature fusion, EMSAM effectively captures both global and local features, highlighting the efficacy of combining CNN and ViT for plant disease segmentation. Models relying solely on ViT, such as MedSAM and Trans-Unet, exhibit lower Dice and IoU scores due to insufficient attention to local features. While DeepLabv3+ achieves an IoU of 0.6504, its ResNet-50 backbone struggles with fine-grained boundary information. Swin-Unet and Trans-Unet show relatively lower performance, indicating room for improvement in segmenting complex disease images. HRNet-48, with Dice and IoU scores of 0.6911 and 0.5643 respectively, demonstrates advantages in multi-scale feature extraction but still falls short of EMSAM’s overall performance. Notably, EMSAM achieves the highest Dice coefficient of 0.7925, surpassing the second-best model, DeepLabv3+, by 2.45%, and outperforming the lowest-performing Swin-Unet by 31.92%. This underscores EMSAM’s superior overall match quality for segmented regions, particularly in boundary detection and small region segmentation. Additionally, its IoU of 0.6987 surpasses DeepLabv3+ and Swin-Unet by 6.91% and 35.84%, respectively.

This study compares EMSAM with SOTA methods in terms of total parameters, learnable parameters, and floating-point operations (FLOPs), as detailed in **Table 3**. EMSAM has a total of 589.6M parameters and 133.9M learnable parameters, demonstrating its ability to capture complex features. Its computational cost of 322.5G FLOPs is higher than that of smaller models such as DeepLabv3+ and Swin-Unet but significantly lower than larger models like MedSAM and Trans-Unet. As a transformer-based model, EMSAM has the highest overall parameter count among these models. However, the adoption of parameter-efficient fine-tuning techniques and lightweight module designs helps keep the number of learnable parameters at a moderate level. Overall, EMSAM achieves a balance between accuracy and model complexity by maintaining sufficient representational capacity while optimizing parameter efficiency and computational cost. This makes it particularly suitable for challenging segmentation tasks involving ambiguous lesion boundaries and diverse morphological variations.

**Table 3:** Comparison of EMSAM and SOTA methods in terms of model parameters, learnable parameters, and training resource consumption.

Method	Total params (M)	Learnable params (M)	FLOPs (G)
SAMUS	514.3	85.3	300.1
MedSAM	357.7	29.3	177.3
DeepLabv3+	209.6	209.6	118.7
Swin-Unet	114.3	114.3	142.9
Trans-Unet	461.2	461.2	492.1
HRNet-48	197.9	197.9	167.2
EMSAM	589.6	233.9	322.5

**Figure 8:**
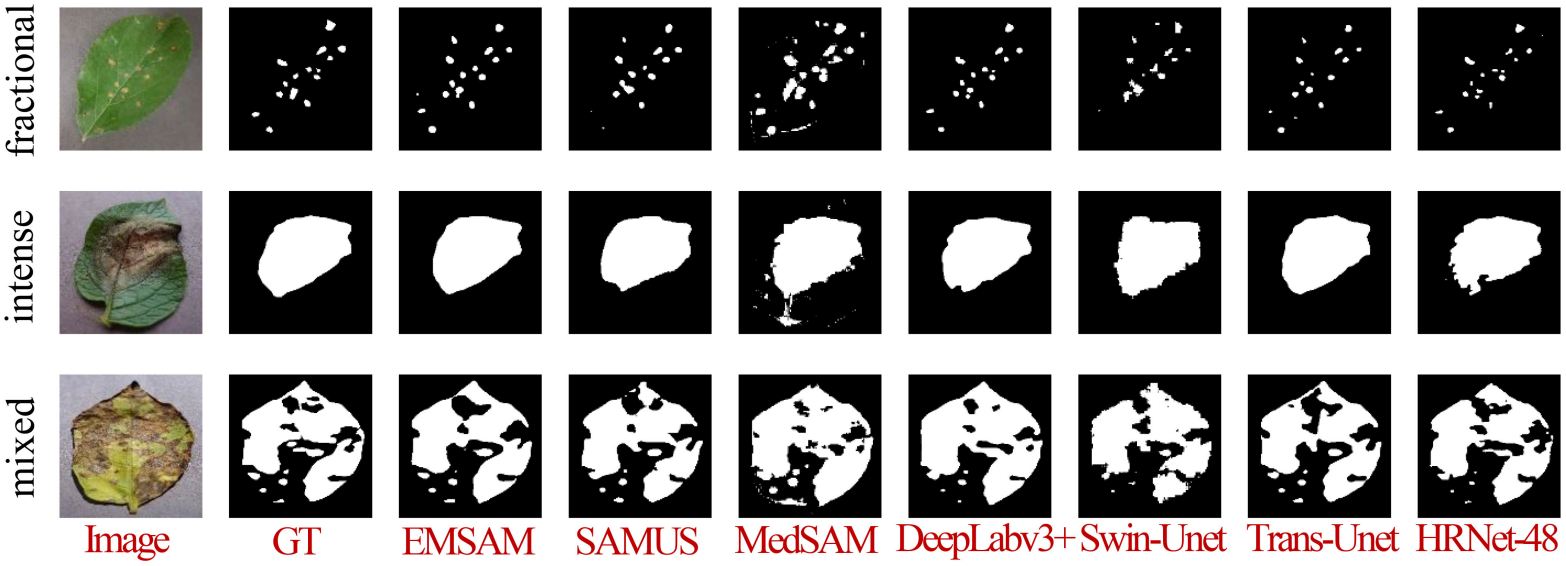
Visualization of different models on the self-constructed the PSD.

**Figure 8** demonstrates that EMSAM achieves refined pixel-level segmentation, effectively capturing both global and local features while maintaining the integrity of disease regions and preserving superior boundary details. Compared to EMSAM, SAMUS exhibits difficulties in detecting small lesions, while MedSAM’s sensitivity to noise results in false boundary delineations. In the first row, representing small and fragmented lesions, EMSAM outperforms the other models with accurate segmentation. In the second row, depicting large and concentrated lesions, EMSAM excels in preserving boundary details, while DeepLabv3+, although robust in segmenting large areas, struggles with fine-grained precision. In the third row, depicting mixed feature distributions, EMSAM accurately segments disease regions while avoiding noise, unlike HRNet-48, which exhibits noticeable lesion adhesion.

### 3.3 Analysis of segmentation ability for different disease severity levels

To validate EMSAM’s adaptability in segmenting leaf diseases across different severity levels, we reclassified the test set based on the disease severity classification method outlined in Section 2.1, categorizing the leaves into light, moderate, and severe severity levels. A detailed analysis of each model’s performance under these conditions is presented in **Table 4**, showing the Dice and IoU scores for different models across the three severity levels.

**Table 4:** Comparison of model performance at three disease levels.

Method	Dice			IoU		
	Light	Moderate	Severe	Light	Moderate	Severe
SAMUS	0.6361	0.7898	0.6979	0.5928	0.6545	0.6536
MedSAM	0.6757	0.6139	0.6446	0.5142	0.5213	0.5047
DeepLabv3+	**0.7564**	0.7759	0.7839	**0.6372**	0.6714	0.6426
Swin-Unet	0.4678	0.4769	0.6738	0.3195	0.3842	0.6412
Trans-Unet	0.6521	0.7354	0.6862	0.4357	0.5306	0.5288
HRNet-48	0.7128	0.6925	0.6682	0.5782	0.5567	0.5579
EMSAM	0.7236	**0.8354**	**0.8187**	0.6254	**0.7365**	**0.7342**

The bold values indicate the optimal data metrics achieved by the model under the current experimental setup.

**Figure 9:**
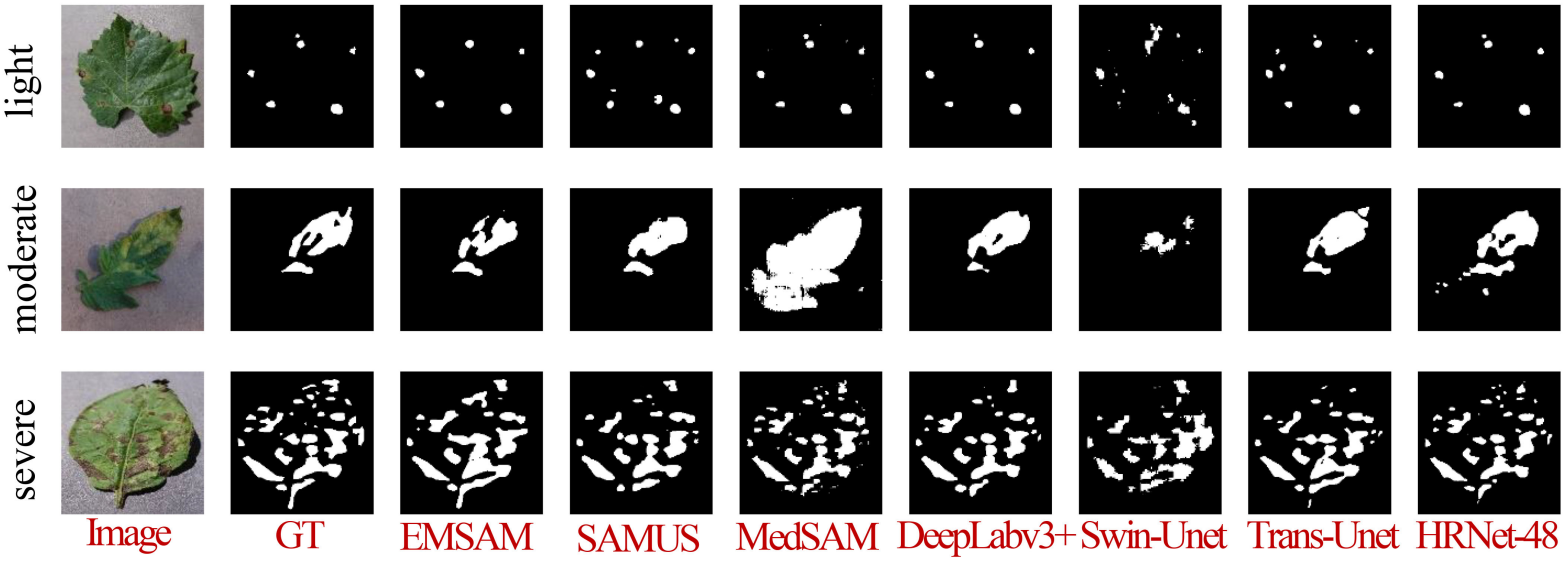
Visualization of the model when confronted with different disease degree classes.

Under light disease conditions, DeepLabv3+ achieves the highest Dice score of 0.7564 and IoU of 0.6372, significantly outperforming other models and demonstrating superior fine feature capture. EMSAM follows closely, exhibiting high stability, while Swin-Unet records the lowest performance with an IoU of 0.3195, indicating challenges in handling small lesion areas. In medium disease conditions, EMSAM achieves Dice and IoU scores of 0.8354 and 0.7365, respectively, outperforming MedSAM, which shows a relatively lower IoU of 0.5213, highlighting limited robustness in moderately complex lesions. For severe disease conditions, EMSAM maintains its leading performance with Dice and IoU scores of 0.8187 and 0.7342, respectively, showcasing strong adaptability and generalization in complex scenarios **Figure 9** presents visual segmentation outcomes across varying disease severity levels, revealing model performance disparities. MedSAM and Swin-Unet, sensitive to foreground-background discrepancies, exhibit poor performance on leaves with ambiguous boundaries. In light disease scenarios, CNN-based models such as DeepLabv3+ and HRNet-48 excel at capturing small lesion areas, while EMSAM demonstrates a more balanced performance. For medium severity cases, EMSAM performs notably well, whereas MedSAM struggles with indistinct lesion boundaries. Under severe disease conditions, Trans-Unet and SAMUS experience over-segmentation due to heightened sensitivity to complex backgrounds, hindering performance improvement.

### 3.4 Impact of different hyperparameter settings on model performance

**Figure 10:**
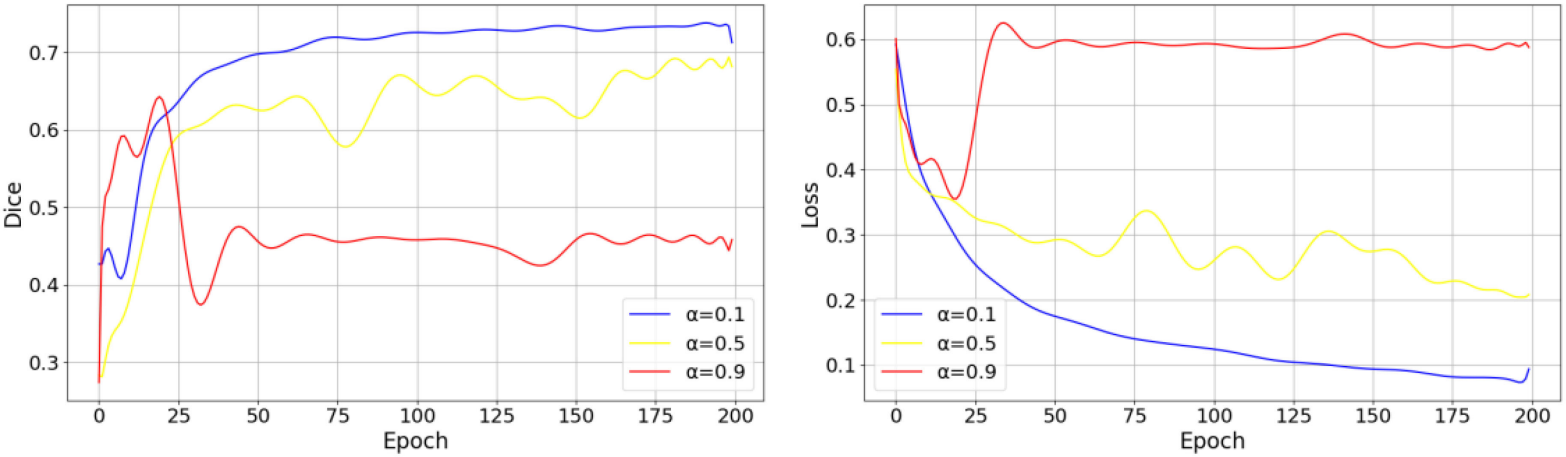
The impact of a fixed $\alpha_{epoch}$ on model performance.

In Section 2.2.3, after introducing the SE Block, we design a dynamic coefficient $\alpha_{epoch}\;$ to control the growth process of Epoch-based Num, as expressed in Equation 20. Additionally, in Section 2.2.1, we introduce a joint loss function ${ℒ}_{joint}$ to simultaneously cater to segmentation and classification tasks, as defined in Equation 21. These equations introduce two new hyperparameters to EMSAM: the growth rate control parameter β and the weights λ for the loss component. To investigate the impact of dynamic and fixed parameter settings on model performance, we designed relevant experiments and visualized key training indicators, as shown in **Figure 10**. The left plot shows the Dice score variation over epochs, while the right plot shows the loss variation over epochs. In this experiment, we selected three values (0.1, 0.5, and 0.9) to control the influence of the SE block on the training process. When $\alpha_{epoch}\;$ =0.1, the SE block has a relatively minor effect on the overall model training, allowing the Dice score to increase rapidly in the early training phase while maintaining relatively high segmentation accuracy in later stages. Moreover, the overall training process remains stable. In contrast, when $\alpha_{epoch}\;$ =0.9, the SE block exerts a much stronger influence on the model training, leading to significantly lower Dice scores compared to other settings. This suggests that an excessively strong influence from the SE block during the early training phase can cause the model to become overly sensitive to its effects, making the optimization process too slow to adapt to later-stage loss variations. Consequently, the model fails to fully leverage the feature representations learned in the early stages, which ultimately compromises performance.

**Figure 11:**
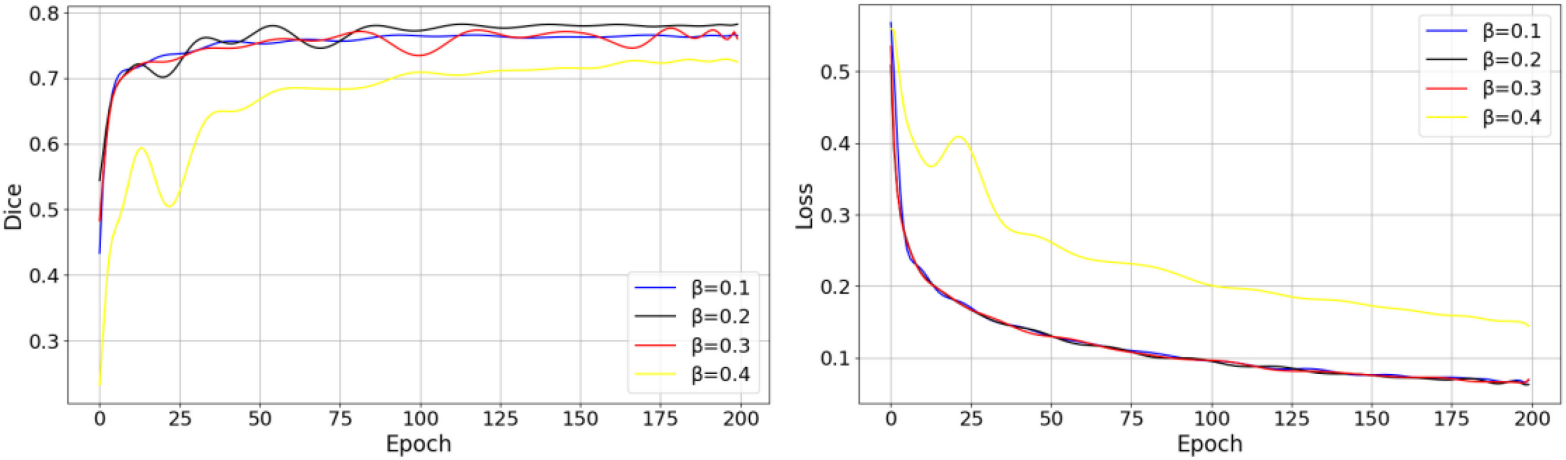
Effect of different hyperparameter settings on the training process.

The experimental results in **Figure 10** indicate that dynamically adjusting the influence of the SE block during training is essential for achieving a better balance between convergence speed and final accuracy. To observe the effects of the dynamic parameter β on model performance during training, we experiment with different values for each and conduct training sessions to identify the appropriate hyperparameter settings. **Figure 11** illustrates the impact of varying β values on the model’s loss function and Dice Coefficient.

As shown in the figure above, different values of β have a significant impact on the model’s early convergence speed and the final stable value. When β=0.4 , the convergence speed is the fastest, but the final loss value is higher than those of other hyperparameter settings, which suggests that the model may converge too quickly, leading to underfitting. In contrast, β=0.1 and β=0.2 exhibit lower final loss values. Considering the effect of different β values on the Dice, β=0.2 contributes more effectively to optimizing the model’s performance, striking a good balance between training efficiency and segmentation performance. **Table 5** presents the effects of different weight settings for the three components of the joint loss function ${ℒ}_{joint}$ on model performance.

**Table 5:** Effect of different Loss weights on model performance.

$\lambda_{mask}$	$\lambda_{IoU}$	$\lambda_{cls}$	Dice	IoU	Acc
0.4	0.3	0.3	0.7059	0.6257	0.8672
0.5	0.3	0.2	0.7433	0.6328	0.8637
0.6	0.3	0.1	0.7813	0.6893	0.7932
0.6	0.2	0.2	**0.7925**	**0.6987**	**0.8786**

The bold values indicate the optimal data metrics achieved by the model under the current experimental setup.

From **Table 5**, it is evident that the weights $\lambda_{mask}$ and $\lambda_{IoU}$ positively influence segmentation performance, while the Acc metric does not exhibit a clear monotonic trend, instead fluctuating based on the weight combinations. Setting $\lambda_{mask}=0.6$ , $\lambda_{IoU}=0.2$ , $\lambda_{cls}=0.2$ effectively balances the weight distribution between segmentation and classification tasks, resulting in notable performance improvements for the model.

### 3.5 Ablation study

In EMSAM, the core components are the proposed MAA, LFEM, and FFM. **Table 6** presents ablation experiment results on the PSD, analyzing the contributions of these components. The baseline model, shown in the first row, employs traditional adapter tuning for SAM transfer (Chen et al., 2023a). The second row incorporates MAA for adapter tuning, validating its effectiveness in extracting multi-scale information. The third row introduces LFEM as an efficient detail feature supplement, utilizing standard cross-modal attention for information fusion. The fourth row further incorporates an SE-based attention mechanism into the information fusion process to prevent detail loss during feature integration.

**Table 6:** Analysis of ablation experiments on the PSD.

MAA	LFEM	FFM	Dice	IoU
✕	✕	✕	0.6117	0.5284
✓	✕	✕	0.7782	0.6824
✓	✓	✕	0.7870	0.6928
✓	✓	✓	0.7925	0.6987

Symbol ✕ represents the baseline configuration excluding the module, whereas symbol ✓ demonstrates that its inclusion induces statistically significant alterations in model performance.

**Figure 12:**
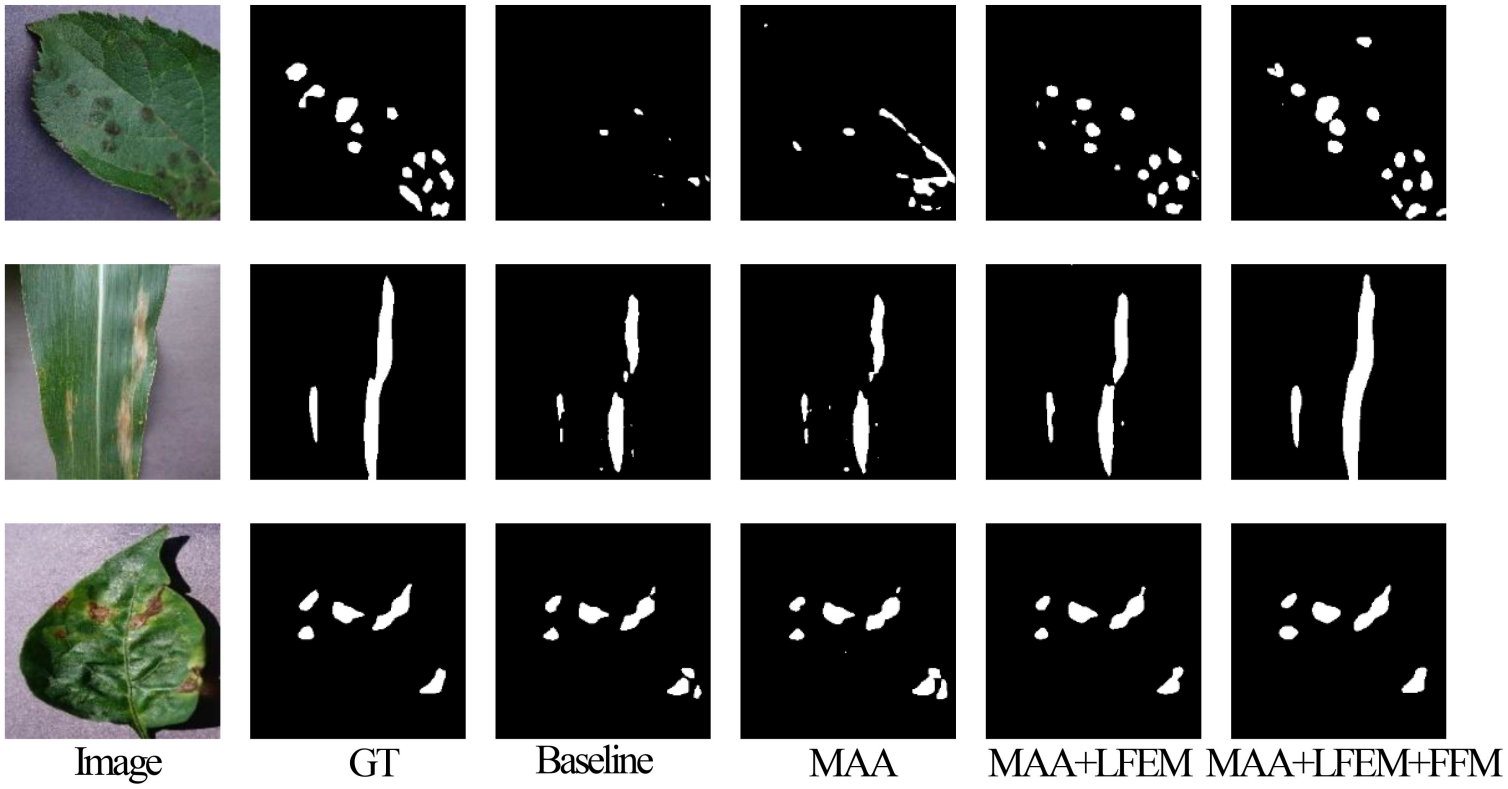
Visualization comparison of EMSAM’s core components.

To illustrate the contributions of EMSAM’s core components, we present a visual analysis of segmentation performance across different model configurations. **Figure 12** provides a visual comparison of segmentation outcomes for EMSAM’s core components: MAA, LFEM, and FFM. The baseline model shows notable limitations in segmenting complex lesion regions, particularly in capturing boundary details and detecting small lesions. Incorporating the MAA module substantially improves segmentation performance, enabling more effective extraction of multi-scale features compared to traditional adapter tuning methods. Adding the LFEM further enhances the model’s ability to process details and boundaries, leading to more precise local feature extraction, particularly in small lesion areas and at lesion edges. Incorporating the FFM module effectively integrates features from the CNN and ViT branches, resulting in the best overall segmentation performance for both lesion area segmentation and boundary detail capture.

The ablation study demonstrates that the introduced components (MAA, LFEM, and FFM) each significantly improve segmentation performance. MAA enhances multi-scale feature extraction in complex lesion regions, LFEM boosts local feature extraction and boundary processing, and FFM facilitates efficient channel-level feature fusion between CNN and ViT branches. Collectively, these modules synergize to elevate the model’s segmentation capabilities, particularly in handling complex and detailed disease regions.

## 4 Conclusion

In this study, we propose EMSAM to address the challenges of blurred boundaries and complex shapes in leaf disease images. Built upon the SAM architecture, EMSAM achieves superior performance over state-of-the-art segmentation algorithms on the PSD, demonstrating robust generalization across varying disease severities and diverse scenarios. The MAA, specifically designed for leaf disease image processing, efficiently captures blurred boundary features through a multi-scale information extraction module. The LFEM employs lightweight convolutional groups to extract fine-grained features, complementing the global information captured by ViT blocks and enabling balanced attention to both global and local features. The FFM uses SE blocks to dynamically balance the weights between CNN and ViT branches, effectively reducing redundancy. Lastly, a lightweight classification head in the decoder integrates segmentation and classification tasks for efficient multi-task learning.

Despite the promising performance of EMSAM in leaf disease segmentation, several limitations need to be acknowledged: (1) The study primarily relies on the PlantVillage dataset, which, while widely used in plant disease research, consists of images captured in controlled environments with simple and uniform backgrounds. This limits the model’s ability to generalize to real-world agricultural settings where lighting variations, occlusions, and complex backgrounds pose additional challenges. Future work should evaluate EMSAM on diverse field datasets to enhance its robustness and applicability. (2) Although EMSAM integrates parameter-efficient tuning techniques, the inclusion of MAA, FFM, and attention mechanisms increases computational overhead compared to standard CNN-based approaches. The additional FLOPs and memory requirements may hinder real-time deployment on edge devices with limited resources. Future optimizations, such as knowledge distillation or pruning, could help reduce inference costs while maintaining performance. To further enhance the capabilities of EMSAM, future research could explore two primary avenues. Firstly, expanding the training dataset to encompass a broader range of plant species and environmental conditions would likely bolster the model’s generalization across diverse scenarios. Secondly, exploring more efficient multi-task learning paradigms could optimize performance on auxiliary tasks without increasing model complexity, thereby enhancing both primary and auxiliary task outcomes. These directions hold the potential to significantly advance the effectiveness and applicability of EMSAM in real-world settings. Overall, EMSAM presents innovative research directions and technical frameworks to enhance image processing in leaf disease segmentation, advancing the development of more efficient and accurate disease detection solutions.

In addition to its demonstrated superiority in leaf disease segmentation, the proposed EMSAM framework exhibits considerable potential for broader real-world applications. The integration of multi-scale adaptive modules and hybrid feature extraction—combining CNN-based local detail capture with ViT-based global context modeling—enables EMSAM to effectively handle images characterized by blurred boundaries and complex object shapes. Consequently, this methodology is not only well-suited for plant disease detection in precision agriculture but can also be readily adapted to other challenging segmentation tasks. For instance, its robust performance in delineating fine structures makes it a promising candidate for medical image analysis (e.g., tumor or lesion segmentation), remote sensing applications (e.g., land cover mapping), and industrial quality control (e.g., defect detection). The modular design and parameter-efficient tuning strategy further facilitate customization for domain-specific requirements, even in scenarios with limited annotated data.

## Data Availability

The datasets presented in this article are not readily available because the dataset is part of ongoing research efforts and cannot be shared publicly until these studies are completed. Requests to access the datasets should be directed to Li Junlong, lijunlong1321@163.com.
